# The Interplay Between CA19‐9 and the Lewis Blood Group System: Implications for the Diagnosis of Gallbladder Cancer

**DOI:** 10.1155/cjgh/8465615

**Published:** 2025-11-14

**Authors:** Yuhan Liu, Jiamao Zhang, Zitong Zheng, Wenjuan Zheng, Lu Fang, Fan Zhou, Bo Liang

**Affiliations:** ^1^ Department of Hepatobiliary Surgery, The Second Affiliated Hospital of Nanchang University, Nanchang, Jiangxi, 330006, China, jxndefy.cn; ^2^ Queen Mary School, Nanchang University, Nanchang, Jiangxi, 330006, China, ncu.edu.cn; ^3^ The Second Clinical Medical School, Nanchang University, Nanchang, Jiangxi, 330006, China, ncu.edu.cn; ^4^ Institute of Translational Medicine, Nanchang University, Nanchang, Jiangxi, 330006, China, ncu.edu.cn

**Keywords:** CA19-9, diagnosis, gallbladder carcinoma, Lewis blood groups

## Abstract

CA19‐9 is one of the most widely applied tumor markers in clinical oncology, particularly in the diagnosis and prognostic evaluation of biliary tract and pancreatic cancers. However, its clinical utility is limited by biological variability, most notably the influence of the Lewis blood group system, which may result in false‐negative findings. The Lewis system is composed of three major phenotypes, Le(a+b−), Le(a−b+), and Le(a−b−), with the less frequent Le(a+b+) also reported in certain populations. Current evidence indicates that CA19‐9 expression occurs predominantly in Le(a+b−) or Le(a−b+) individuals, whereas it is largely absent in Le(a−b−) individuals. Insufficient consideration of this factor in clinical settings reduces diagnostic accuracy and complicates interpretation of CA19‐9 results. This review summarizes the current understanding of the relationship between CA19‐9 and the Lewis system, critically evaluates recent clinical studies, highlights existing limitations, and discusses the potential role of combined CA19‐9 and Lewis typing in improving the diagnostic value of gallbladder cancer.

## 1. Introduction

CA19‐9 is a polyglycan antigen that contains glycoproteins and glycolipids, which was first discovered by Koprowski et al. in 1979 [1]. In the physiological state, circulating CA19‐9 levels in healthy individuals maintain a baseline concentration typically (less than 37 U/mL). While in patients with specific malignancies (such as pancreatic and gallbladder cancer), serum concentrations of CA19‐9 are typically elevated [2, 3]. Extensive research over the years has substantiated the significant clinical value of CA19‐9 in multiple aspects of gallbladder cancer management, including diagnostic evaluation, prognostic assessment, and prediction of tumor resectability [4–8].

However, the clinical utility of CA19‐9 as a diagnostic biomarker is subject to certain limitations. In clinical practice, despite histopathologically confirmed malignancy, some patients have undetectable CA19‐9 levels [9]. This phenomenon may be multifactorial in origin, with a significant contributing factor potentially associated with the individual’s Lewis blood group genotype [10]. Three Lewis blood types are commonly observed in clinical practice, including Le(a+b−), Le(a−b+), and Le(a+b+), while the fourth type, Le(a−b−), is occasionally seen in certain races [11]. Among these, individuals with Le(a+b−), Le(a−b+), and Le(a+b+) blood types can express CA19‐9. In contrast, most individuals with the Le(a−b−) blood type cannot express or only minimally express CA19‐9 due to the absence of fucosyltransferase, an enzyme essential for catalyzing the CA19‐9 precursor [12]. Therefore, the influence of the Lewis blood type should be considered when evaluating the expression level of CA19‐9 in clinical practice. This review mainly discusses the relationship between CA19‐9 and the Lewis blood type to provide insights for improving the diagnosis of gallbladder cancer.

## 2. The Lewis Blood Group System and CA19‐9

### 2.1. The Lewis Blood Group System

The Lewis blood type system, a special blood type system, was first discovered and named by Mourant et al. in 1946. Its antigens are type I glycan structures, which are formed by the adsorption of Lewis substances present in plasma onto the surface of red blood cells [13, 14]. The cellular origin of these antigens is epithelial cells distributed across multiple tissue types, including digestive, respiratory (bronchial), reproductive (mammary and seminal), urinary, and orbital systems. These epithelial cells release glycan antigens through exocrine secretion for external release or interstitial deposition, with subsequent diffusion into the plasma compartment [15]. Currently, six Lewis antigens have been identified, among which the most important are Le^a^ and Le^b^ antigens. These antigens are produced through the enzymatic action of fucosyltransferases encoded by two independent genes, FUT3 and FUT2, on precursor substances. Based on the expression of these antigens, four primary Lewis blood type phenotypes are recognized: Le(a+b+), Le(a+b−), Le(a−b+), and Le(a−b−) [16].

### 2.2. Biosynthesis of CA19‐9 and the Major Lewis Antigens

**Figure 1:**
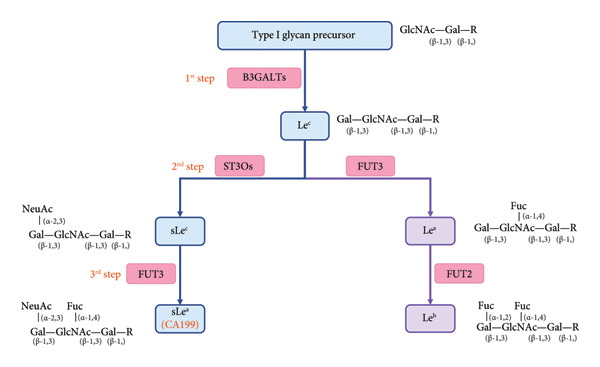
The biosynthesis process of CA19‐9 and major Lewis antigens. B3GALTs, β‐1,3‐galactosyltransferases; ST3O, α‐2,3‐sialyltransferase; FUT3, α‐1,3/4‐fucosyltransferase; FUT2, α‐1,2‐fucosyltransferase.

The biosynthesis of CA 19‐9 in cells occurs through a multistep enzymatic pathway [17]. CA19‐9 is a tetrasaccharide antigen, also known as sialyl Lewis a (sLe^a^), with the sequence Neu5Acα2, 3Galβ1, 3(Fucα1,4) GlcNAcβ1R [18]. As Figure 1 shows, the synthesis of CA19‐9 occurs in major three steps (Figure 1). In the first step, β‐1,3‐galactosyltransferase (B3GALTs) catalyzes the addition of an active galactose residue to the type I carbohydrate chain precursor, forming the Le^c^ antigen. In the second step, α‐2,3‐sialyltransferase (S3TOs) catalyzes the transfer of a sialic acid residue to the GlcNAc portion, connecting it in an α‐2,3 manner to form the sLe^c^ antigen. In the third step, α‐fucosyltransferase (FUT3) encoded by the Le gene catalyzes the binding of an α‐fucose residue to the precursor in an α‐1,4 manner, forming CA19‐9.

The biosynthesis of major Lewis antigens shares the same precursor antigen Le^c^ with CA19‐9. Through the catalytic action of FUT3, the Le^c^ antigen undergoes α‐1,4‐fucosylation to form the Le^a^ antigen. In individuals carrying a functional Se gene, the encoded FUT2 enzyme subsequently catalyzes the α‐1,2‐fucosylation of the Lea antigen, ultimately yielding the Le^b^ antigen [19].

From the above, it is evident that FUT3 plays a crucial role as a shared enzyme in the biosynthesis of both CA19‐9 and Lewis antigens.

### 2.3. The Effect of Lewis Genotype on CA19‐9 Synthesis

Most individuals express minimal amounts of Le^a^ antigen, which usually remain undetectable by conventional phenotypic assays. But rare instances occur where individuals exhibit coexpression of both Le^a^ and Le^b^ antigens, a phenomenon attributable to reduced catalytic efficiency of a functionally compromised FUT2 enzyme [20]. Table 1 illustrates the impact of Lewis genotype on CA19‐9 synthesis. From a mechanistic perspective, individuals with the Le(a+b−) genotype (Le/Le or Le/le with se/se) exhibit normal FUT3 activity but lack FUT2 activity. Consequently, they do not synthesize Le^b^ antigen and only produce Le^a^ antigen, leading to high levels of secreted CA19‐9. In contrast, individuals with the Le (a−b+) genotype (Le/Le or Le/le with Se/Se or Se/se) demonstrate balanced FUT3 and FUT2 activity. Most of the Le^a^ antigen generated is converted to Le^b^ antigen, resulting in moderate levels of secreted CA19‐9. When both Le and Se genes are expressed but the Se gene expression is weak (Se^w^), the FUT2 activity is reduced. This diminishes substrate competition, allowing many Le^a^ antigens to remain unconverted to Le^b^ antigens. As a result, individuals with the Le (a+b+) genotype exhibit high levels of secreted CA19‐9. Conversely, if a Le gene mutation significantly reduces or completely abolishes FUT3 activity, the ability to synthesize Lewis antigens is nearly lost. Thus, individuals with the Le(a−b−) genotype show low levels of secreted CA19‐9 [19].

**Table 1:** The effect of Lewis genotype on the synthesis of CA19‐9.

Lewis blood type	Genotype	FUT2 activity (Se)	FUT3 activity (Le)	CA19‐9 secretion level
Le (a+b+)	LeLe/Lele SeSe/Sese (weak)	Relatively low	Active	High
Le (a+b−)	LeLe/Lele sese	No activity	Active	High
Le (a−b+)	LeLe/Lele SeSe/Sese	Normal	Active	Medium
Le (a−b−)	lele	—	Inactive	Low

*Note:* — indicates independence from FUT2 activity.

## 3. The Statistical Relationship Between CA19‐9 and Individuals With Different Lewis Blood Types

An understanding of the secretion mechanism of CA19‐9 explains the observed distribution patterns among different Lewis blood groups, a phenomenon that has been consistently documented in clinical studies. In a cohort of 433 patients with primary sclerosing cholangitis (PSC), Wannhoff et al. stratified individuals based on FUT2 and FUT3 activity, revealing significant differences in median CA19‐9 levels of cancer‐free patients: 2.0 U/mL (no FUT3 activity), 17.0 U/mL (functional FUT2 and FUT3), and 37.0 U/mL (nonfunctional FUT2 with active FUT3) (*p* < 0.001) [21]. Separately, a research team led by Guo and Luo from Fudan University assessed CA19‐9 levels across different Lewis and Secretor genotypes. Among healthy individuals, median CA19‐9 concentrations were 0.60 U/mL (95% CI, 0.60–0.60) in the Lewis (−) group, 7.49 U/mL (95% CI, 6.83–8.31) in the Lewis (−) Secretor (+) group, and 18.72 U/mL (95% CI, 16.22–20.73) in the Secretor (−) Lewis (+) group (*p*  <  0.001) [10] Another study by the same team found that, within the Han population, the genotypes Le/Le or Le/le combined with Se/Se or Se/se are the most prevalent, accounting for 71.52% of the population. In healthy individuals with these genotypes, serum CA19‐9 levels generally fall within the normal range. In contrast, the Le(a−b−) blood type, which accounts for approximately 10% of the population, is associated with extremely low CA19‐9 levels [22]. In addition, Narimatsu et al. analyzed serum samples from 400 healthy subjects and found that the Le(a+b+) group represented the largest proportion (48.5%) and exhibited relatively stable CA19‐9 levels. In contrast, the Le(a+b−) group showed the highest average serum CA19‐9 content. Meanwhile, the Le(a−b−) group accounted for approximately 10% of the total sample, with serum CA19‐9 levels being undetectable [23].

Researchers further investigated the serum CA19‐9 levels in approximately 10% of the population with the Le(a−b−) blood type under pathological conditions. Parra‐Robert et al. [11] investigated 14 Lewis‐negative cancer patients, of whom 12 patients had CA19‐9 levels close to zero. Narimatsu’s team [23] performed a combined detection of CA19‐9/DU‐PAN‐2 (a cell antibody that is overexpressed in tumor cells and was used for cancer marker detection in the past) on 15 Lewis‐negative colorectal cancer patients, and the results showed that serum CA19‐9 levels were all less than 1.0 unit/mL while another tumor marker DU‐PAN‐2 level exceeded the cutoff value, and even 7 patients had results far higher than Lewis‐positive control group patients. Liu et al. [12] performed a combined detection of CA19‐9/CA125 on 100 Lewis‐negative individuals among 853 pancreatic cancer patients and found that their serum CA19‐9 level was far lower than the normal range, while serum CA125 level exceeded the reference range. Therefore, clinically, for Lewis‐negative patients, in addition to CA19‐9, imaging and other indicators are also of great significance to assist diagnosis [24, 25].

However, in recent years, some clinical data have shown that in a small number of Lewis‐negative patients, serum CA19‐9 levels also increase significantly under pathological conditions. Guopei Luo’s team [26] reported that 27.4% of the samples had serum CA19‐9 levels higher than 37 U/mL among their collected Lewis‐negative pancreatic cancer patients. Yazawa’s team found that 18 Lewis‐negative patients showed the presence of fucosyltransferase in the serum with significantly elevated CA19‐9 levels [27]. Some scholars have put forward their own guesses about this situation: Etsuko’s team studied three such patients with late‐stage cancer of this situation and believed that this may be related to the excessive polysaccharides synthesis as precursors for CA19‐9 and, meanwhile, the certain subtype variation of Lewis recessive blood type may play an important role [28]. The mechanism behind this special increase is not clear yet, and the relationship between Lewis blood type and CA19‐9 synthesis needs further exploration [29].

## 4. Discussion

Serum CA19‐9 levels plays a role in the diagnosis of biliary and pancreatic tumors, particularly in cases of radiographically atypical biliary and pancreatic tumors, as well as ampullary duodenal tumors. However, a subset of patients with clinically suspected tumors exhibit normal CA19‐9 levels, which complicates the diagnostic process. Literature analysis revealed that CA19‐9 expression is closely linked to the Lewis blood group, with patients exhibiting Lewis‐negative tumors showing nearly undetectable CA19‐9 levels [14]. In clinical practice, it is crucial to recognize this phenomenon to avoid false‐negative diagnoses in such patients.

On this basis, in cases where imaging suggests gallbladder cancer, a positive CA19‐9 result increases the likelihood of malignancy. If CA19‐9 is negative, further testing of the Lewis blood group is necessary. When the patient’s Lewis blood type is negative, CA19‐9 may yield a “false‐negative” result and thus lacks diagnostic value. However, if the Lewis blood type is positive, the condition is more likely to be benign. Our previous study indicated that such cases are often pathologically diagnosed as gallbladder polyps or tubular adenomas with dysplasia (Figure s1).

In addition to the Lewis blood group, other factors may also contribute to false‐negative CA19‐9 results. Emerging evidence suggests that distinct tumor subtypes exhibit significant heterogeneity in the expression of glycosylation‐related genes. Given that CA19‐9 biosynthesis requires the coordinated action of specific glycosyltransferases, this molecular heterogeneity may result in the loss of CA19‐9 synthetic capacity in certain subtypes, consequently leading to false‐negative results in serological assays [30]. Besides, analytical interferences including heterophilic antibodies and platform‐specific methodological variations may also lead to falsely low or inconsistent CA19‐9 results, underscoring the importance of method‐specific validation and clinical correlation in the interpretation of CA19‐9 levels [31]. While this study centers on CA19‐9 false negatives, false positives remain a clinically significant concern. Elevated serum CA19‐9 levels have been documented in a wide spectrum of benign conditions, including cholelithiasis [32], cholangitis [33], pancreatitis [34], liver cirrhosis [35], and type 2 diabetes mellitus [36]. These nonmalignant causes of CA19‐9 elevation may substantially compromise the specificity of CA19‐9 as a tumor marker and contribute to diagnostic ambiguity in clinical practice. It should be noted that Lewis antigens are synthesized by epithelial cells and enter the bloodstream via saliva and other secretions rather than being directly produced by red blood cells. Consequently, the phenotype (determined through serum testing) may differ from the genotype due to various influencing factors [37]. In clinical practice, focusing on the genotype can help ensure accurate blood type determination and appropriate consideration in diagnostic decision‐making.

In conclusion, while CA19‐9 retains clinical relevance in gallbladder cancer diagnosis, its utility remains limited. Recent studies have explored that the combined use of CA19‐9 with other tumor markers, such as CEA, CA125, and CA242, may enhance diagnostic sensitivity and specificity [2, 38]. For CA19‐9‐negative patients, Lewis blood group testing can help reduce false negatives and improve accuracy, supporting more individualized management. Ultimately, gallbladder cancer diagnosis should be based on an integrated assessment of clinical history, laboratory findings, and imaging rather than relying on a single biomarker. Future studies exploring multimarker panels and personalized approaches may further refine early detection and risk stratification.

## Conflicts of Interest

The authors declare no conflicts of interest.

## Author Contributions

Yuhan Liu, Jiamao Zhang, and Zitong Zheng contributed to conceptualization, literature screening, and manuscript drafting and editing. Yuhan Liu and Jiamao Zhang prepared the figures and tables. Wenjuan Zheng assisted in additional literature retrieval and manuscript revision. Lu Fang contributed to conceptualization and revision. Bo Liang provided overall supervision and final approval. Fan Zhou supervised the revision and editing. Yuhan Liu and Jiamao Zhang contributed equally and should be regarded as cofirst authors. Yuhan Liu and Jiamao Zhang contributed equally to this work.

## Funding

This work was supported by the National Natural Science Foundation of China (grant no. 82160578) and the Natural Science Foundation of Jiangxi Province, China (grant no. 20212BCJ23024).

## Supporting Information

Figure s1 Imaging data of three patients. A: Case 1, MRI report: uneven thickening and abnormal enhancement of the gallbladder wall; B: Case 2, CT report: gallbladder space‐occupyinglesions; C: Case 3, CT report: narrow base of gallbladder with large mass and abundant blood vessels at the basilar part of gallbladder.

## Supporting information


**Supporting Information** Additional supporting information can be found online in the Supporting Information section.

## Data Availability

The data that support the findings of this study are available from the corresponding authors upon reasonable request.
