# Supplementary material for: The Interplay Between CA19‐9 and the Lewis Blood Group System: Implications for the Diagnosis of Gallbladder Cancer
Source: Can J Gastroenterol Hepatol. 2025 Nov 14;2025:8465615. doi: 10.1155/cjgh/8465615 (PMC12616487; doi:10.1155/cjgh/8465615)
Supplement: Supplementary file 1 — Supporting Information Additional supporting information can be found online in the Supporting Information section. [file CJGH-2025-8465615-s001.docx]

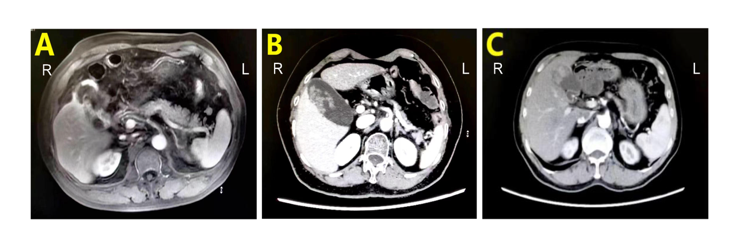


**Figure s1.** **Imaging data of three patients**. A: Case 1, MRI report: uneven thickening and abnormal enhancement of the gallbladder wall; B: Case 2, CT report: Gallbladder Space-occupying Lesions; C: Case 3, CT report: Narrow base of gallbladder with large mass, and abundant blood vessels at the basilar part of gallbladder.
